# Voltage-Gated Potassium Channels Beyond the Action Potential

**DOI:** 10.1089/bioe.2022.0014

**Published:** 2022-05-26

**Authors:** Luis A. Pardo

**Affiliations:** Oncophysiology Group, Max Planck Institute for Multidisciplinary Sciences (MPI-NAT), Göttingen, Germany.

**Keywords:** voltage-gated potassium channels, KCNH1, Kv10.1, electrophysiology, cancer

## Abstract

Bioelectricity goes far beyond electrical signaling in the nervous system, but this was initially not obvious for me. This article describes the journey from studying the biophysics of ion channels in classical electrically excitable tissues to focusing on the pathogenic roles of the Kv10.1 potassium channel in cancers.

## The Difficult Choice Between Bench and Bedside

Very early as a medical student at the University of Oviedo, Spain, I learned the possibility of joining a department and participating in research activities as an internal student, a position designed to offer undergraduates the option to participate in scientific activities. In the 1980s, Spain was making an effort to catch up with modern science, and there were many opportunities for young people to join science.

I was fortunate enough to be admitted as an internal student at the Department of Biochemistry in the group of Sofía Ramos and Pedro Sánchez Lazo. They had built a research team a few years earlier that worked in a field that at the time was starting to bloom, “intracellular signaling.” I could help in laboratory work during lecture-free periods and attend department seminars throughout the year. During the preclinical years, I could manage both enrollments relatively well, but my dedication to basic science became restricted when I started clinical training.

Nevertheless, my contact with the department stayed alive, and so did my passion for experimental science. It was hard to decide whether to continue as a clinician or aim for a Ph.D. after graduating. I believe Sofía's enthusiasm was very contagious and drove me to choose to start a Ph.D. to work on the biochemistry of RAS proteins in yeast. Still very far away from bioelectricity.

During the last months of my Ph.D., Francisco (Paco) Barros, a former Ph.D. student of Pedro and Sofía (who had also taught me some biochemistry courses during my undergraduate studies), had just rejoined as an assistant professor after several years in the United States working on patch-clamp electrophysiology. He was setting up a patch-clamp setup, one of the first ones in Spain. I was immediately fascinated by the power of the technology and the beauty of bioelectrical signals. Hence, when Paco mentioned the possibility of a postdoctoral stay with Walter Stühmer in Göttingen, I was very excited and enthusiastic. And equally scared, because it meant doing a postdoc at the laboratory where patch-clamp had been invented not too long ago (this happened in 1990) knowing essentially nothing about electrophysiology.

## First Electrophysiology Experiences

Somehow it all worked out, and I could join Walter's group, still small at that time. At my arrival, the group was dissecting the structure-function relationships of sodium (and some potassium) channels. During those first months, I had the privilege of witnessing one scientific milestone after the other. The group was integrated into Erwin Neher's department, a collection of outstanding scientists; we enjoyed a very intense and open scientific communication that made work a pleasure and reassured me that my place was being a scientist.

Walter's lab specialized in electrophysiology on *Xenopus* oocytes. We cooperated with molecular biology labs who generated the cRNA of constructs and mutants and performed the electrophysiology experiments, two-electrode, and excised patches from *Xenopus* oocytes. My first electrophysiology project, in close cooperation with Stefan H Heinemann, dealt with RCK4 (now Kv1.4), one of the several *Shaker*-related channels that had been cloned in the laboratory of Olaf Pongs in Hamburg. Kv1.4 was at the time the only known mammalian K^+^ channel showing fast inactivation; it expressed well in oocytes and was an excellent starting point for a newcomer. We could measure relatively big currents in outside-out macropatches, and this system was optimal to study with high temporal resolution the modulation of channel kinetics by extracellular factors.

**FIG. 1.**
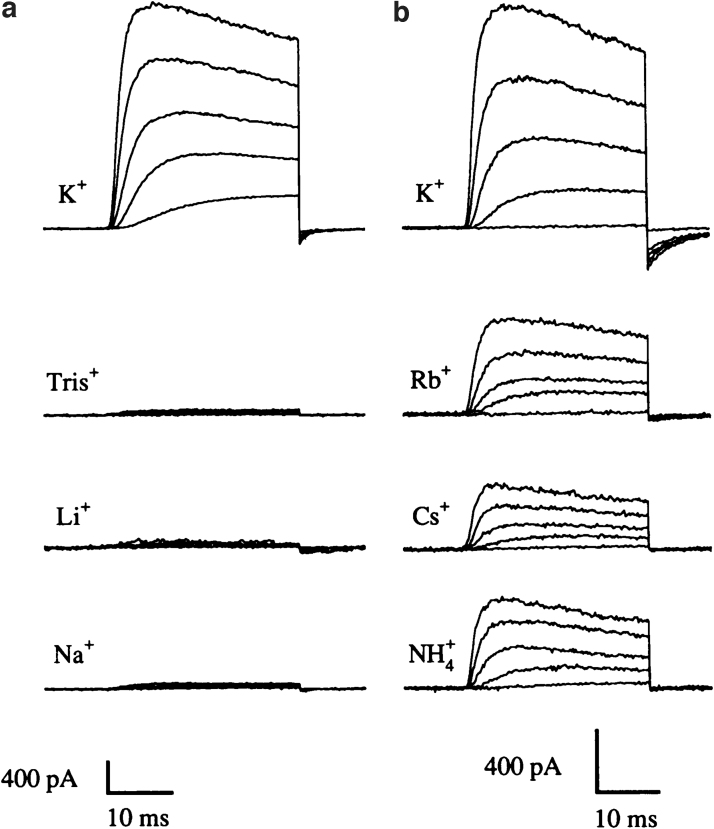
The dependence of Kv1.4 current amplitude on the cations present in the external solution. Currents were measured in outside-out patches from oocytes upon depolarizations between −40 and +40 mV (20 mV steps) from a holding potential of −100 mV. **(a, b)** Obtained from two different patches. The current amplitude is dramatically reduced in the presence of Tris^+^, Li^+^ or Na^+^. 110 mM Tris^+^ was present in all solutions, plus 10 mM of the chloride salt of the indicated ions. Reproduced from Pardo et al.^1^

When changing the extracellular concentration of ions, we noticed that increasing the extracellular concentration of K^+^ resulted in larger currents instead of the smaller amplitudes we expected after reducing the driving force for K^+^. In other words, the decrease in driving force originated by rising potassium concentration outside was compensated by some other phenomenon.^1^ We could not think of many alternatives to explain this behavior. Changes in the inactivation properties, faster recovery from inactivation, and probably an increase in single channel conductance had to occur. We then started reducing rather than increasing the extracellular K^+^ concentration to characterize this better. Partial substitution of K^+^ by Na^+^ rendered, as expected, smaller currents (Fig. 1), but it was remarkable that the current would disappear irreversibly when we removed the extracellular K^+^ entirely.

This effect had been already described on squid axons^2^ but had not been reproduced on any cloned channel whose structure we could manipulate. We identified the site responsible for the unusual sensitivity of Kv1.4 and set out afterward to clarify the mechanism of action.

As part of the collaboration with the laboratory of Olaf Pongs, where the Kv1.4 mutants were generated, we received a visiting student who had cloned the *Drosophila eag* potassium channel and wanted to test whether it formed a functional K^+^ channel. Other laboratories had cloned it before, but it did not seem to produce functional channels.^3,4^ The plan was to test whether we observed any functional expression and, if so, to characterize the major biophysical properties of *eag*. The project was intended to take a few weeks to complete and would be the final part of the Diploma thesis of Andrea Brüggemann, but we were proved dramatically wrong in our expectations. The project filled Andrea's Ph.D. work and continued well beyond that. Thirty years later, I am still working on the channel.

## Kv10.1 and Its Role in the Nervous System

**FIG. 2.**
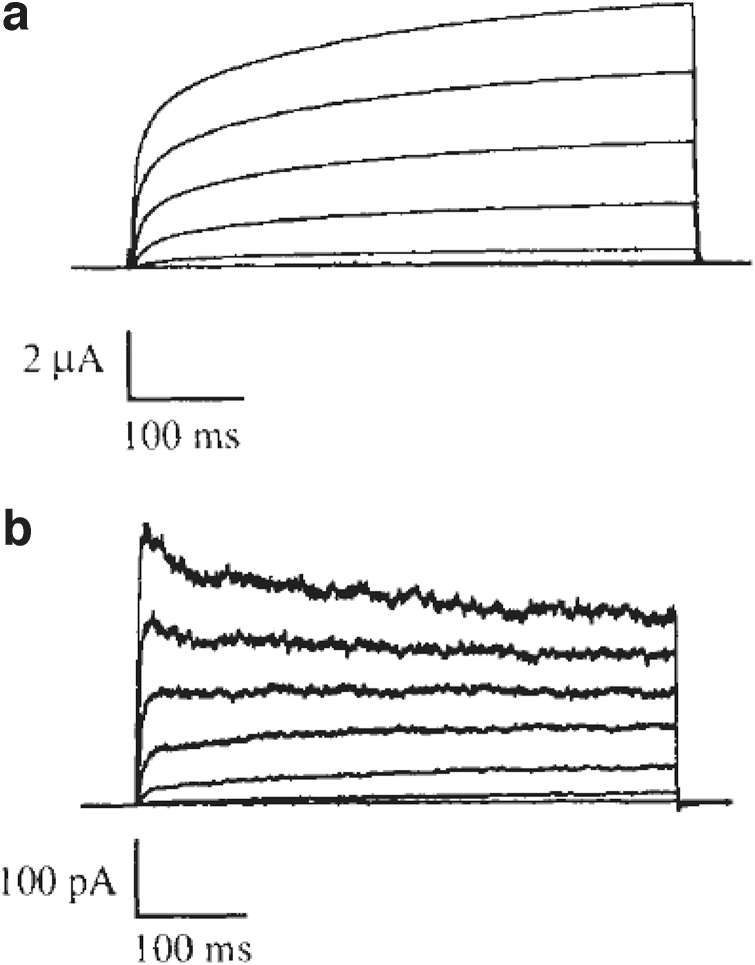
Changes in current kinetics between two-electrode voltage clamp **(a)** and inside-out patches **(b)** from *Xenopus* oocytes expressing the *Drosophila eag* channel. The traces represent the response to depolarizations between −60 and +60 mV. Reproduced from Bruggemann et al.^5^

*D. melanogaster eag* is the founding member of a complex gene family (*KCNH)*, conserved through the *Animalia* kingdom. This channel, in particular, turned out to be a fascinating molecule from the very beginning. First, it showed different kinetics in two-electrodes and excised patches^5^ (Fig. 2) and our first efforts tried to clarify the reasons for this. Admittedly, we never entirely did. We hypothesized that the differences between the two recording modes were due to Ca^2+^-activated Cl^−^ currents endogenous to the oocyte. We suspected that either the channel was permeable to Ca^2+^ or its activation facilitated Ca^2+^ rise in the oocyte. Other laboratories expressed well-founded doubts about this explanation.^6^

Still, the conflicting results were based on a channel construct that behaved identically in oocytes and excised patches, clearly different from the one we had studied. We started a systematic mutagenesis study to address this, but soon the mammalian ortholog was cloned, and we then, thinking that we would find the answers to the questions posed by the fly channel, shifted our efforts to the rat one, whose behavior was not identical to the insect one but still very interesting.

During the initial characterization of the mammalian channel, a kinetic property caught the attention of Heinrich Terlau, at the time a senior postdoc at Walter's lab. He was puzzled by the observation that the activation of the channel at one potential was sometimes fast and sometimes markedly slower.^7,8^ Cole and Moore^9^ had described an apparently similar phenomenon (but probably mechanistically different^10^) many years before. This is why the effect was (and often still is) termed the Cole-Moore shift.

The speed of activation of Kv10.1 depends markedly on the potential before the stimulus, providing a sort of “molecular memory” to the channel. When the starting point is a hyperpolarized potential like in a resting neuron, Kv10.1 takes milliseconds to begin activating and is therefore irrelevant during an action potential. But with successive stimuli, since the terminal has been depolarized, the activation is much faster.

This explains why, as Sünke Mortensen showed using a knockout mouse generated by Roser Ufartes, the parallel-fiber/Purkinje cell synapses of knockout mice^11^ underwent a frequency-dependent increase in facilitation and enhanced Ca^2+^-influx into axonal boutons upon repeated stimuli,^12^ leading to a certain degree of hyperexcitability only detectable under stress conditions. This can be seen as the primary neurophysiological role of Kv10.1 channels.

## Electromechanical Coupling in Kv10.1 (and Other *KCNH* Channels)

With its peculiar behavior, understanding at a molecular level the gating of Kv10.1 has taken a significant part of our attention. The structural data provided by MacKinnon's laboratory on *KCNH* channels^13–15^ brought crucial information, and we now understand the mechanisms governing electromechanical coupling in the family much better, although not yet completely.

Some years ago, the consensus opinion was that the action of the S4–S5 segment of the channel as a rigid loop was a fundamental part of the gating of all voltage-gated channels, transmitting the movement of the voltage sensor to the gate. We assumed that this was also true for *KCNH* channels, but we were ignoring the fact that looking at the hydropathy plots and alignments with channels with known structure, the length of the S4–S5 segment in Kv10.1 was probably too short to allow for a “mechanical lever mechanism,” as the cryo-EM structure later confirmed.

**FIG. 3.**
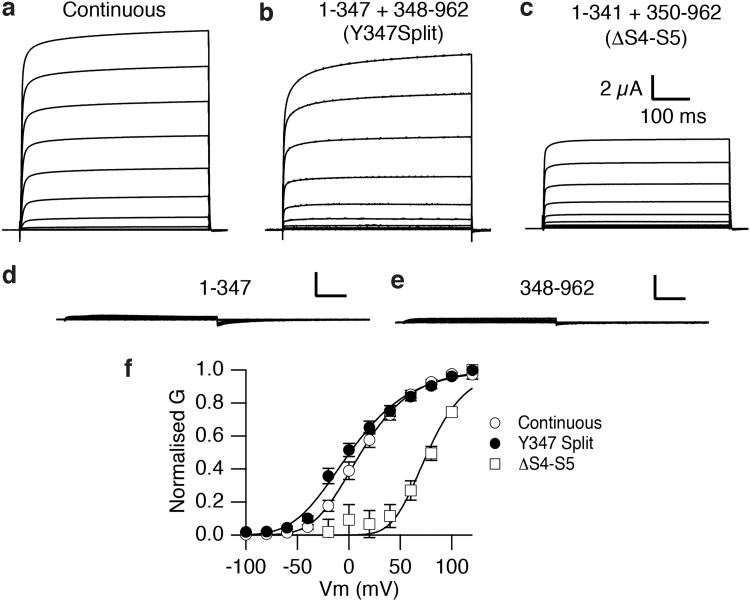
Voltage-dependent currents from discontinuous Kv10.1 channels. Current traces obtained by depolarizations (−80 to +80 mV at 20 mV intervals) in oocytes injected with **(a)** cRNA for continuous Kv10.1, **(b)** a mixture of cRNAs encoding for residues 1–347 and 348–962 or **(c)** residues 1–341 and 350–962 (S4–S5 deletion). All gave rise to outward currents. **(d, e)** No current is detected using the same stimuli as in **(a–c)** in 60 mM extracellular K^+^ in oocytes injected with cRNA encoding 1–347 (left) or 348–962 (right) Kv10.1. **(f)** Conductance/voltage plot of continuous (open circles), 347-split (closed circles) and split Kv10.1 channels lacking the S4–S5 linker (open squares). The split channel lacking the S4–S5 segment required stronger depolarizations to respond, but its voltage dependence (the slope of the GV curve) was not dramatically different. Adapted from Lörinczi et al.,^16^ under Creative Commons Attribution 4.0 International License.

It also revealed that the domain swapping observed in Kv1 (*KCNA*) channels, which is essential to transfer the movement of the voltage sensor to the relevant parts of the pore domain, was not present in *KCNH* channels. While performing a series of controls to generate chimeric channels, Eva Lörinczi generated two constructs encoding each an independent protein, one extending from the amino terminus to the end of S4 (or what we believed at that time was the S4–S5 segment) and another one starting there and extending until the end of the C-terminus. Against our expectations, the spontaneous assembly of those proteins in *Xenopus* oocytes formed a voltage-gated channel (Fig. 3), allowing us to rule out the possibility that the S4–S5 segment acts as a mechanical lever^16,17^ in this and other KCNH channels.^18^ Still, the alternative mechanisms are incompletely understood.

## K_V_10.1 and the Cell Cycle

While initially studying the ion selectivity of the rat Kv10.1, we noticed that Cs^+^ gave rise to measurable currents through the channel. Cs^+^ is the ion classically used to avoid contamination by K^+^ currents because the selectivity filter excludes it. This observation indicated that *eag* channels were much less selective for K^+^ than other channels.

This was an exciting phenomenon, especially after our observations on the fly channel, and we thought we could easily quantify the selectivity of Kv10.1 expressing the channel in Chinese hamster ovary cells and performing whole-cell patch-clamp measurements. However, the experiments turned out very challenging because cell variability was remarkable. After a good patch-clamp session with many successful recordings, when we thought that we had quantified the selectivity, things could suddenly stop working, and a new dish of cells would show no permeability to Cs^+^.^19^ Such a change in channel behavior had never been observed (or to a lesser extent) in oocytes.

After a long (and frustrating) search, we came to the idea that a very relevant difference between *Xenopus* oocytes and somatic cells is that cultured cells can be at any phase of the cell cycle, but preferentially at G1 simply because that is the most prolonged phase, but oocytes are physiologically arrested at the G2 phase of the first meiotic division.

Luckily, it is possible both to synchronize cells in different phases of the cycle and to induce progression of the cycle in oocytes with relative easiness. We tested both things and could document that the properties of the channel are profoundly altered as the cell cycle progresses.^19,20^ At that point, we showed that “mitosis promoting factor” (the complex between CDK1 and cyclin B in oocytes) modifies the properties of the channel profoundly, and that at different phases of the cycle, the current looked different. However, we still did not have a mechanistic explanation for this observation.

At about the same time, we noticed that it was challenging to obtain good cell densities for patch-clamp experiments on Kv10.1-transfected cells. Of the several cell lines expressing heterologous channels that we run in parallel for electrophysiology, only Kv10.1 cells were systematically too dense. It was hard to find isolated cells for patching. Under other circumstances, we would have probably just downscaled the cell density for Kv10.1 measurements and moved forward. Yet, because we had been dealing with the cell cycle, we came to the idea that it could be that the interaction of Kv10.1 with the cell cycle is bidirectional. If Kv10.1 is regulated by cell cycle progression. In this case, it is plausible that the channel also plays a role in controlling the cell cycle and that the cycle is accelerated in cells overexpressing the channel. This was indeed the case, and we started thinking that the phenomenon might have physiological consequences.

Up to this point, we had been working with the rat channel. We then set out to clone the human ortholog because a protein participating in cell cycle modulation in human cells could be relevant for physiopathology. We started studying the possible effects of manipulating Kv10.1 expression or function in the human breast cancer cell line MCF7. Halima Ouadid-Ahidouch also proposed functional expression of the channel in these cells in Natalia Prevarskaya's laboratory.^21^

We decided to screen the cell line for Kv10.1 cDNA, and Donato del Camino and Araceli Sánchez could identify a fragment with a sequence almost identical to the rat Kv10.1. Using that fragment as a probe, they started screening a human mammary gland library in a frustrating process with no success. Before entirely giving up, they gave a last try to the probe on a human brain library, where they could very rapidly obtain a full-length clone of the human channel. Later on, we understood that the channel is not detectable in the normal mammary gland because the expression profile of the channel is rather specific to the brain, and therefore the screening was not successful.

## Kv10.1 in Cancer

This simple observation had profound consequences. We had a channel virtually absent from normal peripheral tissues, but that can be detected in tumor cell lines, is regulated by the cell cycle, and can accelerate the proliferation of cells. These are all features that would make it a good candidate for a factor relevant to tumor progression and amenable to therapeutic targeting. Animal experiments confirmed that tumors expressing Kv10.1 grew faster and were more aggressive than control tumors.^22^ Was this observation in animals relevant for human cancer?

Rüdiger Weseloh, who had produced antibodies against Kv10.1 during his Ph.D., joined the lab and designed monoclonal antibodies that we could use to detect the presence of the channel in human samples. He teamed up with Bernhard Hemmerlein, a pathologist at the Pathology department of the University Hospital across the road. Using the new antibodies, they screened many breast cancer samples and found that a large majority of tumors expressed significant amounts of Kv10.1. Surprisingly, this was true not only for breast cancer but also for all the types of cancer we could test at the time.^23^ Notably, the presence of Kv10.1 in tumors was not an epiphenomenon since inhibiting the expression of Kv10.1 slowed down consistently the proliferation of tumor cells.^24^

In summary, the channel is undetectable in normal peripheral tissues, expressed in a majority of tumors, and its downregulation or inhibition can reduce the growth of the tumor. This immediately led to the hypothesis that Kv10.1 inhibitors could offer an alternative strategy to treat many cancers, alone or in combination. We had little doubt what the ethically acceptable decision was; we have attempted to translate this hypothesis into a clinical reality using many different strategies. Still, we constantly faced three problems that we continue striving to solve.

First, direct inhibition of Kv10.1 using pharmacological intervention (either small molecules or antibodies) never destroyed the experimental tumors in animals, and it only was able to slow down their growth.^25^ This is a relative problem, but in practical terms, it makes further developments difficult, among other reasons, because determining the endpoint parameters that can be measurable in a feasible time for clinical trials becomes challenging. To overcome this difficulty, we have designed some strategies that take advantage of the selective expression of the channel in tumors and, rather than inhibiting the channel's function, deliver cytokines (mainly TRAIL) to the tumor cells.

**FIG. 4.**
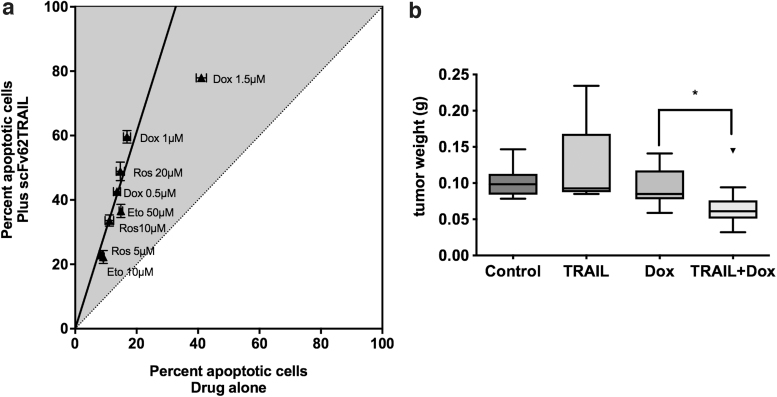
Induction of apoptosis on MDA-MB435S cells by scFv62-TRAIL (single chain anti-Kv10.1 antibody fused to TRAIL). **(a)** Plotting percent apoptosis in the presence of scFv62-TRAIL (0.1 μg/mL) and the indicated drugs (etoposide, cisplatin, roscovitine, paclitaxel, doxorubicin) for 18 h versus apoptosis induction in the absence of the construct highlights the synergistic effect, which was comparable for all drugs and linearly correlated to the concentration of the drug. Apoptosis was determined by Annexin V staining. **(b)** *In vivo* efficacy of scFv62-TRAIL (0.15 mg/kg) in combination with doxorubicin (0.9 mg/kg). Tumor weight was determined *ex vivo* after six treatment cycles. Adapted from Hartung and Pardo,^30^ under Creative Commons Attribution 4.0 International License.

This approach combines two sources of specificity (TRAIL, TNF-related apoptosis-inducing ligand, which is preferentially active against tumor cells,^26^ and Kv10.1, which is preferentially expressed in those cells). It improves the efficacy of TRAIL because it mimics the membrane-bound form of the protein.^27^ For the strategy to work, it is imperative that the construct does not reach the nervous tissue since TRAIL can induce the death of astrocytes and Kv10.1 is present in normal neurons.^28^ In any case, several combinations of antibodies against Kv10.1 and different forms of TRAIL have proven efficacious *in vitro* and *in vivo*,^29–31^ and enhance the action of conventional chemotherapeutics (Fig. 4).

A second problem is the structural similarity between Kv10.1 and Kv11.1 (HERG). Kv11.1 is the paradigm of an *antitarget*, and every candidate drug is tested to discard action on Kv11.1 due to potential cardiotoxicity.^32^ Advances in the knowledge of the structure of both channels and computer-assisted drug design methods can help find truly specific drugs.^33^ A certain level of cross-reactivity with Kv11.1 could be a manageable problem under the control of a competent cardiologist. Still, because we expect such drugs to only retard tumor growth and possibly impede metastasis, they would very likely be used in combination with other agents and, therefore, in patients whose general status is already compromised. Both factors would increase the complexity of the treatment, and the search for specific compounds is still justified.^34^

The third problem is the lack of a precise mechanism by which Kv10.1 facilitates tumor growth and worsens the outcome of patients. The mechanistic uncertainty increases the risk of unexpected adverse events and, therefore, the development cost. Since the first phenomenological descriptions, our knowledge has improved substantially. Manipulation of the expression levels or function of Kv10.1 alters many of the hallmarks of cancer, affecting cell growth, epithelial to mesenchymal transition,^35^ cell adhesion,^36^ migration,^37^ cell resistance to hypoxia and angiogenesis,^38^ mitochondrial dynamics,^39^ and cytoskeletal function,^40^ but the molecular mechanisms leading to such manifold consequences are incompletely understood.

An important observation that has conceptual implications is that Kv10.1, although normally not detected in non-neural tissues, is in fact expressed by many cell types, but only during a short time in the life of a given cell, namely during the G2/M transition of the cell cycle.^41^

The expression of the channel is under the control of the transcription factor E2F1,^42^ a master regulator of the cell cycle^43^; at the same time, several non-coding RNAs regulate channel expression,^44–46^ increasing the complexity of the system. Thus, the expression of Kv10.1 is inhibited at different levels in normal cells. When the cell commits to divide, E2F1 is activated, and it binds the promoter of the channel after some hours, at the time of the initiation of mitosis. Then, the expression is turned on, and the channel is detectable on the cell surface of the cell. This may explain the correlation between papillomavirus infection and Kv10.1 overexpression.^47,48^

Nevertheless, the expression levels are never massive, and the subcellular localization of the channel at the plasma and other membranes plays a significant role. The efforts to characterize the subcellular localization of the protein gave us an example of the power of electrophysiological techniques. We observed that the nuclear envelope of cells transfected with green fluorescent protein-labeled channels systematically lighted robustly up. This could be an artifact because of overexpression but could also be a true localization of the channel. We performed immunocytochemistry experiments, electron microscopy, and subcellular fractionation, and all those approaches indicated localization in the inner nuclear membrane.

**FIG. 5.**
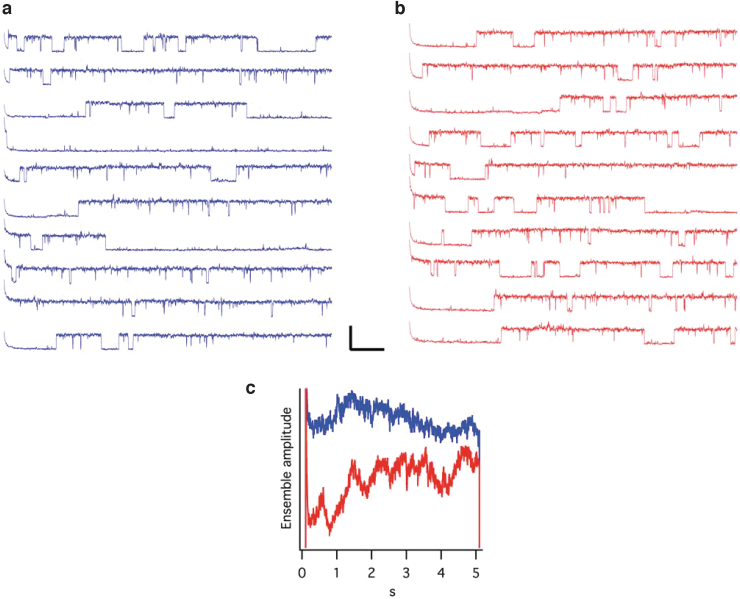
Single channel recording from the inner nuclear membrane of HEK-Kv10.1 cells. The activity was recorded at +60 mV from a holding potential of −60 **(a, blue)** or −100 mV **(b, red)**. The time before the first opening is increased when the holding potential is more negative (pseudo Cole-Moore shift). This can be better appreciated in the ensemble currents depicted **(c)**. Scale bars: 2 pA, 500 ms. Adapted from Chen et al.^49^ under Creative Commons Attribution 4.0 International License.

All of those approaches have pros and cons, and they depend on antibodies; although we could confirm the results with several different antibodies, we still needed solid proof of the identity of the channel. Fortunately, Kv10.1 is, after all, an ion channel, so it should be possible to measure its activity in case it was functional at this location. We set out to measure single channels in preparations of nuclei devoid of the outer membrane and could document the presence of channel activity with selectivity, kinetics, and pharmacology compatible with Kv10.1. Probably the most convincing argument is the kinetic one because of the very peculiar behavior of this channel (Fig. 5).

During pulse and chase experiments, we made the surprising observation that at least part of the channels located at the internal nuclear membrane had been exposed to the extracellular milieu at the plasma membrane just 30 min earlier.^49^ Is this a way to communicate external events directly to the nucleus in cells? Why would a large protein like this ion channel fulfill such a task that could be done by smaller soluble messengers? Is potassium flow influencing, for example, the formation of G quadruplexes, important for the modulation of transcription^50^? These questions remain to be answered.

We still try to understand the exact molecular mechanisms responsible for the more aggressive phenotype of tumors expressing Kv10.1. What are the advantages that those cells have? A lot of the evidence we have collected stems from studying the changes that knocking it down induces in the cells. These are very complex and possibly context-dependent, but some shared features suggest a general mechanism of action. As a rough summary, Kv10.1 participates in cytoskeletal-related functions in a Ca^2+^-dependent fashion. Among other observations, mitochondrial dynamics and microtubule assembly change when Kv10.1 is either knocked down or inhibited in tumor cells; both processes depend critically on Ca^2+^ signaling, and Ca^2+^ oscillations are abnormal when Kv10.1 is inhibited or knocked down.^39,40^

Intuitively, it is easy to imagine a situation where a K^+^ channel modulates Ca^2+^ entry just by controlling the resting membrane potential. Again, this hypothesis is probably too simple, and besides the canonical function of Kv10.1 as a channel, it plays other roles, probably at a structural level.

In non-tumor cells, knockdown of Kv10.1 prevents the resorption of the primary cilium when the cell cycle progresses, which is bound to many changes in signal transduction mechanisms.^51^ Knockdown cells display a primary cilium under conditions that should prevent ciliation. The timing of ciliary resorption matches the time window when Kv10.1 is expressed, and repression of ciliation is a common feature of many cancers, so ciliogenesis is an excellent candidate to underlie many of the consequences of dysregulated expression. But this particular role of the channel also highlights the existence of both canonical and non-canonical mechanisms related to the physiology of Kv10.1.

**FIG. 6.**
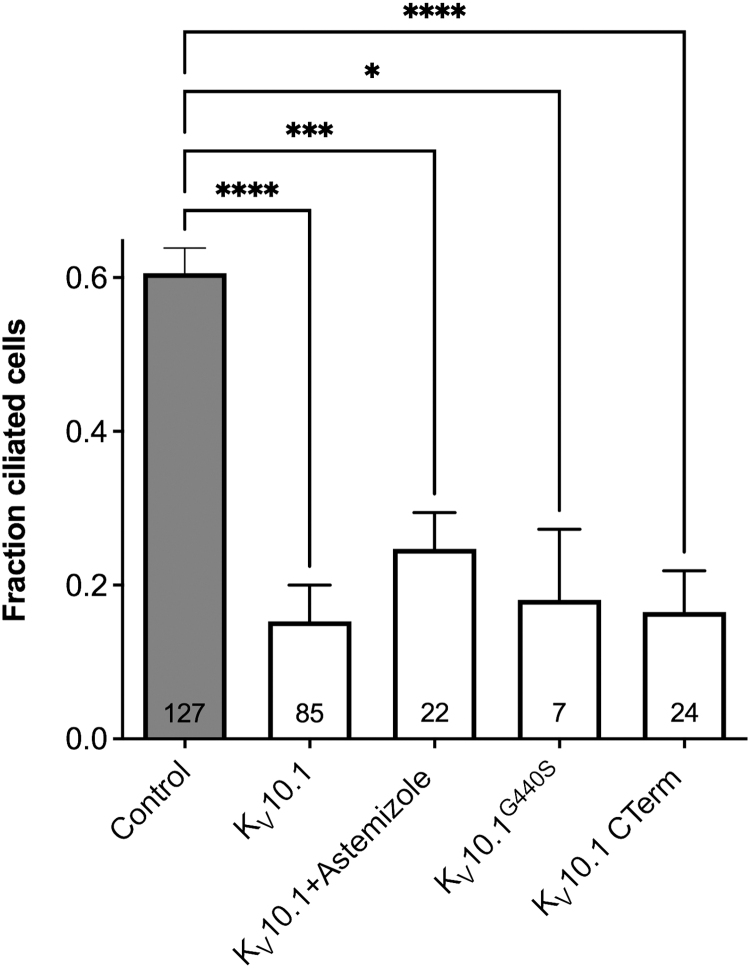
Kv10.1 overexpression reduced the frequency of ciliated cells in cells where ciliation was stimulated by serum starvation. The reduction of the number of ciliated cells was not dependent on potassium permeation. The effect was similar when K^+^ permeation was inhibited by 10 μM astemizole, when a non-permeant mutant channel (G440S) was used, or when only the intracellular C-terminus was transfected. Bars represent mean ± SEM, and the numbers in the bars indicate the number of images analyzed. The asterisks indicate *p*-values (all conditions were significantly different from the control (ANOVA). The figure is adapted from panel A of Fig EV3 in Sánchez et al.,^51^ under a Creative Commons license; the order of the bars has been altered to adapt it to the current text. ANOVA, analysis of variance; SEM, standard error of the mean.

Overexpression of the whole channel has the opposite effect to knockdown, and cells will not respond to treatments promoting ciliation, such as serum starvation. Still, our initial thought that this was just the reversal of the same effect proved (again) wrong because part of the effects can be reproduced when only the C-terminus of the channel is overexpressed, in the absence of the pore domains and therefore of ion permeation. In the control of ciliary resorption, the canonical function induces the effects, but the C-terminus of the channel alone prevents ciliation (Fig. 6). Therefore, permeation of K^+^ through Kv10.1 is an essential part of the signal. Still, interactions with other partners in a supramolecular complex that likely requires the presence of the C-terminus of Kv10.1 to form or stabilize are also important.

## Concluding Remarks

In summary, the interest in ion channel biophysics led us to a journey toward cell and tumor biology that is still far from its end. The specific features of ion channels and the availability of techniques with unparalleled resolution make it possible to achieve a deep understanding of the molecular mechanisms underlying the physiology and pathophysiology and should help transforming the translation into clinical benefit for the patients from a promising perspective into a reality. Maybe the journey leads us back to the bedside.

## References

[1] Pardo LA, Heinemann SH, Terlau H, et al. Extracellular K+ specifically modulates a rat brain K+ channel. Proc Natl Acad Sci U S A 1992;89:2466–2470. 1549610 10.1073/pnas.89.6.2466 PMC48679

[2] Almers W, Armstrong CM. Survival of K+ permeability and gating currents in squid axons perfused with K+-free media. J Gen Physiol 1980;75:61–78. 7359118 10.1085/jgp.75.1.61 PMC2215183

[3] Warmke J, Drysdale R, Ganetzky B. A distinct potassium channel polypeptide encoded by the *Drosophila eag* locus. Science 1991;252:1560–1562. 1840699 10.1126/science.1840699

[4] Zhong Y, Wu C-F. Alteration of four identified K+-currents in drosophila muscle by mutations in eag. Science 1991;252:1562–1564. 2047864 10.1126/science.2047864

[5] Bruggemann A, Pardo LA, Stuhmer W, et al. Ether-a-go-go encodes a voltage-gated channel permeable to K+ and Ca2+ and modulated by cAMP. Nature 1993;365:445–448. 7692301 10.1038/365445a0

[6] Robertson GA, Warmke JW, Ganetzky B. Potassium currents expressed from drosophila and mouse eag cdnas in xenopus oocytes. Neuropharmacology 1996;35:841–850. 8938715 10.1016/0028-3908(96)00113-x

[7] Ludwig J, Terlau H, Wunder F, et al. Functional expression of a rat homologue of the voltage gated either a go-go potassium channel reveals differences in selectivity and activation kinetics between the Drosophila channel and its mammalian counterpart. EMBO J 1994;13:4451–4458. 7925287 10.1002/j.1460-2075.1994.tb06767.x PMC395377

[8] Terlau H, Ludwig J, Steffan R, et al. Extracellular Mg2+ regulates activation of rat eag potassium channel. Pflügers Archiv Eur J Physiol 1996;432:301–312. 8662307 10.1007/s004240050137

[9] Cole KS, Moore JW. Potassium ion current in the squid giant axon: Dynamic characteristic. Biophys J 1960;1:1–4. 13694549 10.1016/s0006-3495(60)86871-3 PMC1366308

[10] Hoshi T, Armstrong CM. The cole-moore effect: Still unexplained? Biophys J 2015;109:1312–1316. 26445430 10.1016/j.bpj.2015.07.052 PMC4601067

[11] Ufartes R, Schneider T, Mortensen LS, et al. Behavioural and functional characterization of Kv10.1 (Eag1) knockout mice. Hum Mol Genet 2013;22:2247–2262. 23424202 10.1093/hmg/ddt076 PMC3652421

[12] Mortensen LS, Schmidt H, Farsi Z, et al. Kv10.1 opposes activity-dependent increase in Ca^2+^ influx into the presynaptic terminal of the parallel fibre-Purkinje cell synapse. J Physiol 2015;593:181–196. 25556795 10.1113/jphysiol.2014.281600 PMC4293062

[13] Whicher JR, MacKinnon R. Structure of the voltage-gated K+ channel Eag1 reveals an alternative voltage sensing mechanism. Science 2016;353:664–669. 27516594 10.1126/science.aaf8070 PMC5477842

[14] Wang W, MacKinnon R. Cryo-EM structure of the open human ether-à-go-go-related K+ channel hERG. Cell 2017;169:422–430.e410. 28431243 10.1016/j.cell.2017.03.048 PMC5484391

[15] Whicher JR, MacKinnon R. Regulation of Eag1 gating by its intracellular domains. Elife 2019;8: e49188. 31490124 10.7554/eLife.49188 PMC6731095

[16] Lörinczi É, Gómez-Posada JC, de la Peña P, et al. Voltage-dependent gating of KCNH potassium channels lacking a covalent link between voltage-sensing and pore domains. Nat Commun 2015;6:6672. 25818916 10.1038/ncomms7672 PMC4389246

[17] Tomczak AP, Fernández-Trillo J, Bharill S, et al. A new mechanism of voltage-dependent gating exposed by K_V_10.1 channels interrupted between voltage sensor and pore. J Gen Physiol 2017;149:577–593. 28360219 10.1085/jgp.201611742 PMC5412533

[18] de la Pena P, Dominguez P, Barros F. Gating mechanism of Kv11.1 (hERG) K(+) channels without covalent connection between voltage sensor and pore domains. Pflugers Arch 2018;470:517–536. 29270671 10.1007/s00424-017-2093-9 PMC5805800

[19] Pardo LA, Bruggemann A, Camacho J, et al. Cell cycle-related changes in the conducting properties of r-eag K+ channels. J Cell Biol 1998;143:767–775. 9813096 10.1083/jcb.143.3.767 PMC2148139

[20] Bruggemann A, Stuhmer W, Pardo LA. Mitosis-promoting factor-mediated suppression of a cloned delayed rectifier potassium channel expressed in Xenopus oocytes. Proc Natl Acad Sci U S A 1997;94:537–542. 9012819 10.1073/pnas.94.2.537 PMC19548

[21] Ouadid-Ahidouch H, Le Bourhis X, Roudbaraki M, et al. Changes in the K^+^ current-density of MCF-7 cells during progression through the cell cycle: Possible involvement of a h-ether.a-gogo K^+^ channel. Recept Channels 2001;7:345–356. 11697078

[22] Pardo LA, del Camino D, Sanchez A, et al. Oncogenic potential of EAG K^+^ channels. EMBO J 1999;18:5540–5547. 10523298 10.1093/emboj/18.20.5540 PMC1171622

[23] Hemmerlein B, Weseloh RM, de Queiroz FM, et al. Overexpression of Eag1 potassium channels in clinical tumours. Mol Cancer 2006;5:41. 17022810 10.1186/1476-4598-5-41 PMC1621079

[24] Weber C, de Queiroz FM, Downie BR, et al. Silencing the activity and proliferative properties of the human EagI Potassium Channel by RNA Interference. J Biol Chem 2006;281:13030–13037. 16537547 10.1074/jbc.M600883200

[25] Gómez-Varela D, Zwick-Wallasch E, Knötgen H, et al. Monoclonal antibody blockade of the human Eag1 potassium channel function exerts antitumor activity. Cancer Res 2007;67:7343–7349. 17671204 10.1158/0008-5472.CAN-07-0107

[26] Walczak H, Miller RE, Ariail K, et al. Tumoricidal activity of tumor necrosis factor-related apoptosis-inducing ligand in vivo. Nat Med 1999;5:157–163. 9930862 10.1038/5517

[27] Naval J, de Miguel D, Gallego-Lleyda A, et al. Importance of TRAIL molecular anatomy in receptor oligomerization and signaling. Implications for cancer therapy. Cancers (Basel) 2019;11:444. 30934872 10.3390/cancers11040444 PMC6521207

[28] Nakamura M, Rieger J, Weller M, et al. APO2L/TRAIL expression in human brain tumors. Acta Neuropathol 2000;99:1–6. 10651020 10.1007/pl00007399

[29] Hartung F, Stuhmer W, Pardo LA. Tumor cell-selective apoptosis induction through targeting of K(V)10.1 via bifunctional TRAIL antibody. Mol Cancer 2011;10:109. 21899742 10.1186/1476-4598-10-109 PMC3179451

[30] Hartung F, Pardo LA. Guiding TRAIL to cancer cells through Kv10.1 potassium channel overcomes resistance to doxorubicin. Eur Biophys J 2016;45:709–719. 27350552 10.1007/s00249-016-1149-7 PMC5045482

[31] Hartung F, Krüwel T, Shi X, et al. A novel anti-Kv10.1 nanobody fused to single-chain TRAIL enhances apoptosis induction in cancer cells. Front Pharmacol 2020;11:686. 32528281 10.3389/fphar.2020.00686 PMC7246340

[32] Sanguinetti MC, Mitcheson JS. Predicting drug-hERG channel interactions that cause acquired long QT syndrome. Trends Pharmacol Sci 2005;26:119–124. 15749156 10.1016/j.tips.2005.01.003

[33] Toplak Z, Hendrickx LA, Abdelaziz R, et al. Overcoming challenges of HERG potassium channel liability through rational design: Eag1 inhibitors for cancer treatment. Med Res Rev 2022;42:183. 33945158 10.1002/med.21808

[34] Toplak Ž, Merzel F, Pardo LA, et al. Molecular dynamics-derived pharmacophore model explaining the nonselective aspect of KV10.1 pore blockers. Int J Mol Sci 2021;22:8999. 34445705 10.3390/ijms22168999 PMC8396485

[35] Restrepo-Angulo I, Sanchez-Torres C, Camacho J. Human EAG1 potassium channels in the epithelial-to-mesenchymal transition in lung cancer cells. Anticancer Res 2011;31:1265–1270. 21508374

[36] Toral C, Mendoza-Garrido ME, Azorin E, et al. Effect of extracellular matrix on adhesion, viability, actin cytoskeleton and K+ currents of cells expressing human ether a go-go channels. Life Sci 2007;81:255–265. 17586530 10.1016/j.lfs.2007.05.014

[37] Hammadi M, Chopin V, Matifat F, et al. Human ether à-gogo K+ channel 1 (hEag1) regulates MDA-MB-231 breast cancer cell migration through Orai1-dependent calcium entry. J Cell Physiol 2012;227:3837–3846. 22495877 10.1002/jcp.24095

[38] Downie BR, Sanchez A, Knotgen H, et al. Eag1 expression interferes with hypoxia homeostasis and induces angiogenesis in tumors. J Biol Chem 2008;283:36234–36240. 18927085 10.1074/jbc.M801830200 PMC2606018

[39] Hernandez-Resendiz I, Pacheu-Grau D, Sanchez A, et al. Inhibition of Kv10.1 channels sensitizes mitochondria of cancer cells to antimetabolic agents. Cancers (Basel) 2020;12:920. 32283712 10.3390/cancers12040920 PMC7226288

[40] Movsisyan N, Pardo LA. Kv10.1 regulates microtubule dynamics during mitosis. Cancers (Basel) 2020;12:2409. 32854244 10.3390/cancers12092409 PMC7564071

[41] Urrego D, Movsisyan N, Ufartes R, et al. Periodic expression of Kv10.1 driven by pRb/E2F1 contributes to G2/M progression of cancer and non-transformed cells. Cell Cycle 2016;15:799–811. 27029528 10.1080/15384101.2016.1138187 PMC4845928

[42] Lin H, Li Z, Chen C, et al. Transcriptional and post-transcriptional mechanisms for oncogenic overexpression of ether à go-go K^+^ channel. PLoS One 2011;6:e20362. 21655246 10.1371/journal.pone.0020362 PMC3105031

[43] van den Heuvel S, Dyson NJ. Conserved functions of the pRB and E2F families. Nat Rev Mol Cell Biol 2008;9:713–724. 18719710 10.1038/nrm2469

[44] Bai Y, Liao H, Liu T, et al. MiR-296-3p regulates cell growth and multi-drug resistance of human glioblastoma by targeting ether-a-go-go (EAG1). Eur J Cancer 2013;49:710–724. 22999387 10.1016/j.ejca.2012.08.020

[45] Wu X, Zhong D, Gao Q, et al. MicroRNA-34a inhibits human osteosarcoma proliferation by downregulating ether a go-go 1 expression. Int J Med Sci 2013;10:676–682. 23569431 10.7150/ijms.5528 PMC3619116

[46] Li Z, Fu Y, Ouyang W, et al. Circ_0016347 promotes osteosarcoma progression by regulating miR-1225-3p/KCNH1 axis. Cancer Biother Radiopharm 2021. DOI: 10.1089/cbr.2019.3349. 33764794

[47] Diaz L, Ceja-Ochoa I, Restrepo-Angulo I, et al. Estrogens and human papilloma virus oncogenes regulate human ether-a-go-go-1 potassium channel expression. Cancer Res 2009;69:3300–3307. 19351862 10.1158/0008-5472.CAN-08-2036

[48] Hwang SG, Lee DY, Kim JY, et al. Human papillomavirus type 16 E7 binds to E2F1 and activates E2F1-driven transcription in a retinoblastoma protein-independent manner. J Biol Chem 2002;277:2923–2930. 11713253 10.1074/jbc.M109113200

[49] Chen Y, Sánchez A, Rubio ME, et al. Functional Kv10.1 channels localize to the inner nuclear membrane. PLoS One 2011;6:e19257. 21559285 10.1371/journal.pone.0019257 PMC3086910

[50] Tateishi-Karimata H, Kawauchi K, Sugimoto N. Destabilization of DNA G-quadruplexes by chemical environment changes during tumor progression facilitates transcription. J Am Chem Soc 2018;140:642–651. 29286249 10.1021/jacs.7b09449

[51] Sánchez A, Urrego D, Pardo LA. Cyclic expression of the voltage-gated potassium channel KV10.1 promotes disassembly of the primary cilium. EMBO Rep 2016;17:708–723. 27113750 10.15252/embr.201541082 PMC5341513

